# Investigating the Association between Coronary Artery Disease and the Liver Fibrosis-4 Index in Patients Who Underwent Coronary Computed Tomography Angiography: A Cross-Sectional Study

**DOI:** 10.3390/jcdd10070301

**Published:** 2023-07-16

**Authors:** Tetsuo Hirata, Yuhei Shiga, Kohei Tashiro, Sara Higashi, Tetsuro Tachibana, Yuto Kawahira, Yasunori Suematsu, Takashi Kuwano, Makoto Sugihara, Shin-ichiro Miura

**Affiliations:** 1 Department of Cardiology, Fukuoka University School of Medicine, Fukuoka 814-0180, Japan; t.hirata.lp@adm.fukuoka-u.ac.jp (T.H.); yuheis@fukuoka-u.ac.jp (Y.S.); kohei.t1027@gmail.com (K.T.); sara3034@icloud.com (S.H.); standflower.22@icloud.com (T.T.); y.kawahira.jo@adm.fukuoka-u.ac.jp (Y.K.); ysuematsu@fukuoka-u.ac.jp (Y.S.); tkuwano1977@gmail.com (T.K.); msma93msma93@yahoo.co.jp (M.S.); 2 Department of Internal Medicine, Fukuoka University Nishijin Hospital, Fukuoka 814-8522, Japan

**Keywords:** coronary artery disease, fibrosis-4 index, hypertension, coronary computed tomography angiography

## Abstract

Liver fibrosis scores, indicative of hepatic scarring, have recently been linked to coronary artery disease (CAD). We investigated the association between CAD and the fibrosis-4 index (FIB-4I) in patients who underwent coronary computed tomography angiography (CCTA). This study included 1244 patients who were clinically suspected of having CAD. The presence or absence of CAD was the primary endpoint. FIB-4I was higher in the CAD group than in the non-CAD group (1.95 ± 1.21 versus [vs.] 1.65 ± 1.22, *p* < 0.001). FIB-4I was also higher in the hypertension (HTN) group than in the non-HTN group (1.90 ± 1.32 vs. 1.60 ± 0.98, *p* < 0.001). In all patients, high FIB-4I (≥2.67) was a predictor of presence of CAD (odds ratio [OR]: 1.92, 95% confidence interval [CI]: 1.30–2.83, *p* = 0.001), and low FIB-4I (≤1.29) was proven to be a predictor of absence of CAD (OR: 0.65, 95% CI: 0.48–0.88, *p* = 0.006). In the HTN group, high and low FIB-4I levels, were found to be predictors for CAD (OR: 2.01, 95% CI: 1.26–3.21, *p* < 0.001 and OR: 0.65, 95% CI: 0.45–0.94, *p* < 0.022, respectively), in particular. FIB-4I may serve as a diagnostic indicator of the presence or absence of CAD in hypertensive patients undergoing CCTA.

## 1. Introduction

Coronary artery disease (CAD) is primarily caused by atherosclerosis [1]. The fluctuating progression of CAD results in a variety of clinical manifestations, which can be classified as acute coronary syndromes (ACS) or chronic coronary syndromes (CCS). CAD is a serious condition that lowers life-expectancy, thereby making the correct diagnosis is important [2]. Numerous risk factors have been identified for CAD, which include hypertension (HTN), dyslipidemia (DL), diabetes mellitus (DM), smoking, family history of cardiovascular disease (FH), chronic kidney disease (CKD), and metabolic syndrome (MetS) [3]. Several risk score systems that combine these coronary risk factors to predict the 10-year prognosis of patients have been established, such as the Framingham Risk Score, Atherosclerotic Cardiovascular Disease score, and Reynolds Risk Score [4]. The lack of other factors may be considered a reason for insufficient prognosis prediction.

Recently, liver fibrosis has garnered attention. Several studies have supported the concept that liver fibrosis is associated with MetS and insulin resistance [5]. Moreover, a positive correlation has been demonstrated between liver fibrosis and CAD in patients with nonalcoholic fatty liver disease (NAFLD) [6]. Cardiovascular diseases have been reported to be the leading cause of death in patients with NAFLD, suggesting a strong association between hepatic fibrosis and cardiovascular disease [7]. Thus, the liver fibrosis index, a standard indicator of the extent of hepatic scarring, may be positively associated with cardiovascular pathologies such as onset and/or progression of CAD. The fibrosis-4 index (FIB-4I), a representative index of liver fibrosis, was originally proposed as a predictive score for liver fibrosis to avoid high-risk liver biopsies in patients with human immunodeficiency virus and hepatitis C virus infection [8]. Subsequently, FIB-4I has been used as a simple index for evaluating the degree of fibrosis in patients with NAFLD. Guidelines also recommend measuring FIB-4I as a non-invasive biochemical predictive method for the diagnosis and management of NAFLD [9].

Coronary computed tomography angiography (CCTA) is a useful diagnostic method for determining the presence and severity of coronary artery stenosis [10]. We previously reported from the Fukuoka University Hospital CCTA (FU-CCTA) registry that HTN, high-density lipoprotein cholesterol (HDL-C), and coronary calcification are predictive factors for CAD [11,12,13]. However, the diagnostic value of FIB-4I in patients who undergo CCTA for CAD screening remains unclear. Therefore, we aimed to investigate whether FIB-4I is associated with CAD using data from the FU-CCTA registry.

## 2. Materials and Methods

### 2.1. Ethics Statement

This study was conducted in accordance with the Declaration of Helsinki, and approved by the Institutional Ethics Committee of Fukuoka University (approval number: #09-10-02, 28 October 2009).

### 2.2. Study Design and Population

This cross-sectional study included 1244 consecutive patients who were clinically suspected of having CAD or who had at least one cardiac risk factor. All patients underwent CCTA between April 2012 and May 2021. Patients with a creatinine level > 2.0 mg/dL or those with contrast-induced allergies did not undergo CCTA. FIB-4I was derived using the following formula at the time of CCTA [8]: [Age (years) × AST (IU/L)]/[PLT count (10^9/L) × √ALT (IU/L)], where AST stands for aspartate transaminase, PLT for platelet, and ALT for alanine transaminase. Determination of CAD was considered as the primary endpoint. Patients were subsequently stratified into CAD and non-CAD clusters according to the presence or absence of HTN.

We evaluated coronary stenosis using CCTA as previously described [14,15,16,17]. A total of 266 patients underwent multidetector computed tomography (MDCT) scans using a 64-MDCT Aquilion-64 machine (TOSHIBA, Tokyo, Japan), whereas 978 patients underwent MDCT using a 320-MDCT Aquilion ONE VISION device (TOSHIBA). Administration of β-blockers and nitroglycerin before scanning was determined on the basis of the attending physician’s judgment. During the initial MDCT scan, a 70-mL injection of contrast medium (Omnipaque, 350 mg iodine/mL; Daiichi Sankyo Co., Ltd., Tokyo, Japan) was administered at a flow rate of 3.6 mL/s. This was subsequently followed by administration of 35 mL of contrast agent and 30 mL of saline solution, both of which were dispensed at a rate of 1.8 mL/s using a dual injector. During the secondary MDCT procedure, a dose of contrast medium equivalent to the patient’s body weight multiplied by 0.7 mL (Iopamiron, 370 mg iodine/mL; Bayer Yakuhin. Ltd., Osaka, Japan) was administered at a rate of 21.5 mgI/kg/s over a duration of 10 s. This was subsequently followed by 35 mL of contrast agent and 30 mL saline solution, both of which were dispensed at a rate of 1.8 mL/s using a dual injector. Scanning was commenced when the computed tomography (CT) density within the region of interest, located in the ascending aorta, registered a value of 100 Hounsfield Units above the baseline CT density. The area from the tracheal bifurcation to the diaphragm was scanned using the following parameters: for 64-MDCT—collimation width of 0.5 mm, rotation speed of 0.4 s/rotation, tube voltage of 135 kV, and effective tube current of 360 mA; for 320-MDCT—collimation width of 0.5 mm, rotation speed of 0.275 s/rotation, tube voltage of 120 kV, and automated tube current. Fifteen segments of the coronary artery were evaluated for each participant. A ≥50% reduction of the normal contrast-enhanced lumen, identifiable through multiplanar reconstructions or axial images, was designated as substantial stenosis in CAD. The severity of coronary atherosclerosis was evaluated using the Gensini score [18].

CT scans were performed using MDCT and a workstation on a Ziostation (courtesy of Ziosoft Inc., Tokyo, Japan).

### 2.3. Data Collection

Information regarding coronary risk factors were collected for all participants. These included FH (myocardial infarction [MI], angina pectoris, or sudden death), smoking status (current or past versus nonsmokers), body mass index (BMI), systolic blood pressure (SBP), diastolic blood pressure (DBP), fasting glucose level, hemoglobin A1c (HbA1c) value, serum levels of total cholesterol (TC), triglycerides (TG), HDL-C, and low-density lipoprotein cholesterol (LDL-C), ratio of LDL-C to HDL-C (L/H), non-HDL-C (TC minus HDL-C), and medication use. BMI was calculated as weight (kg) divided by the square of height in meters (m)^2^. Blood pressure (BP) was determined as the mean of two measurements obtained in an office setting using the conventional cuff method with a mercury sphygmomanometer after at least 5 min of rest. All blood samples were drawn in the morning after the patients had fasted overnight, including a sample for the assessment of liver biochemistry parameters (AST, ALT, and PLT values). Patient demographic data, such as age and coronary risk factors, were retrieved from their electronic medical records. Patients who had a current SBP/DBP ≥ 140/90 mmHg or who were receiving anti-hypertensive therapy were considered to have HTN. Patients with an LDL-C level ≥ 140 mg/dL, TG level ≥ 150 mg/dL, and/or HDL-C level < 40 mg/dL or who were receiving lipid-lowering therapy were considered to have DL. DM was defined using the American Diabetes Association criteria [19] or when the patient was being administered a glucose-lowering drug. MetS in Japan is diagnosed on the basis of the modified guidelines as visceral fat area ≥100 cm^2^ and the presence of two or more of the following: high BP (SBP ≥ 130 mmHg or DBP ≥ 85 mmHg or taking an anti-hypertensive drug), DL (TG level ≥ 150 mg/dL or HDL-C level > 40 mg/dL), or high fasting glucose level (fasting glucose ≥ 110 mg/dL or taking a glucose-lowering drug) [20].

### 2.4. Statistical Analysis

Continuous variables are presented as mean ± standard deviation. Categorical and continuous variables were compared between the groups using the chi-square analysis and *t*-test, respectively. For nonparametric multiple comparisons, we employed the Kruskal-Wallis and Steel-Dwass tests. A multivariate analysis using logistic regression was performed to analyze independent variables (FIB-4I and conventional risk factors) that were related to the presence or absence of CAD. Statistical analyses were performed using Bell Curve for Excel (Social Survey Research Information Co., Ltd., Tokyo, Japan). Statistical significance was set at *p* < 0.05.

## 3. Results

Table 1 shows the characteristics of entire study population (1244 patients), which consisted of 51% females and 49% males. The average age and BMI of the study population were 66 ± 12 years and 24.0 ± 3.8 kg/m^2^, respectively. The prevalences of CAD, HTN, DL, DM, smoking, FH, CKD, and MetS in the entire study population were 50%, 66%, 69%, 25%, 34%, 23%, 29%, and 25%, respectively. The mean AST, ALT, PLT, and FIB-4I values were 27 ± 17 IU/L, 25 ± 21 IU/L, 229 ± 64 × 10^3^/μL, and 1.80 ± 1.22, respectively. Significant differences were observed in the patient characteristics between the CAD and non-CAD groups. The CAD group were significantly older; significantly more likely to be male; had a significantly higher prevalence of HTN, DM, DL, and MetS; significantly higher SBP, fasting glucose, HbA1c, TG, HDL-C, L/H, and FIB-4I levels; and significantly lower HDL-C levels than the non-CAD group. In the context of medications in all patients and in the CAD and non-CAD groups, the percentages of the use of angiotensin II receptor blockers (ARBs)/angiotensin-converting enzyme inhibitors (ACE-Is), calcium channel blockers (CCBs), β-blockers, and statins in all patients were 36%, 39%, 9%, and 31%, respectively. Significant differences were noted in terms of medication use between the CAD and non-CAD groups. The CAD group showed significantly higher use of ARBs/ACEIs, CCBs, β-blockers, statins, sulfonylureas (SUs), biguanides, and dipeptidyl peptidase-4 inhibitors (DPP-4Is) than the non-CAD group.

**Figure 1:**
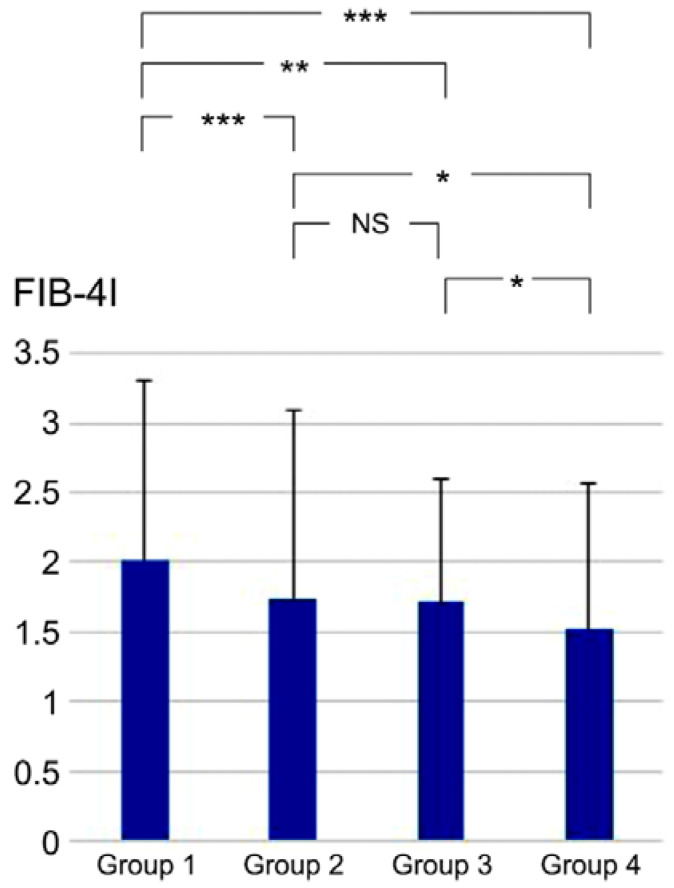
Comparative evaluation of FIB-4I values between the four groups. * *p* < 0.05, ** *p* < 0.01, and *** *p* < 0.001, according to the Kruskal-Wallis and Steel-Dwass tests. Group 1, HTN and CAD; Group 2, HTN without CAD; Group 3, without HTN with CAD; Group 4, without HTN and CAD; NS, not significant; CAD, coronary artery disease; HTN, hypertension; FIB-4I, fibrosis-4 index.

Differences in the FIB-4I values between the CAD and non-CAD groups were assessed and analyzed (Table 2). The FIB-4I level was significantly higher in the CAD group than in the non-CAD group (1.95 ± 1.21 versus [vs.] 1.65 ± 1.22, *p* < 0.001). The FIB-4I level was also significantly higher in the HTN group than in the non-HTN group (1.90 ± 1.32 vs. 1.60 ± 0.98, *p* < 0.001). Furthermore, both the HTN and non-HTN groups in the CAD group had higher FIB-4I values than their non-CAD counterparts. The patients were divided into four distinct groups based on the presence or absence of HTN and CAD (Group 1: HTN and CAD; Group 2: HTN without CAD; Group 3: without HTN with CAD; Group 4: without HTN and CAD). Comparative evaluation of FIB-4I between the four groups was conducted using the Kruskal-Wallis and Steel-Dwass tests, which yielded the outcomes illustrated in Figure 1. A statistically significant difference was observed between Groups 1 and 2 (*p* < 0.001), Groups 1 and 3 (*p* = 0.009), Groups 1 and 4 (*p* < 0.001), Groups 2 and 4 (*p* = 0.014), and Groups 3 and 4 (*p* = 0.020). However, no statistically significant difference was detected between Groups 2 and 3 (*p* = 0.954).

**Figure 2:**
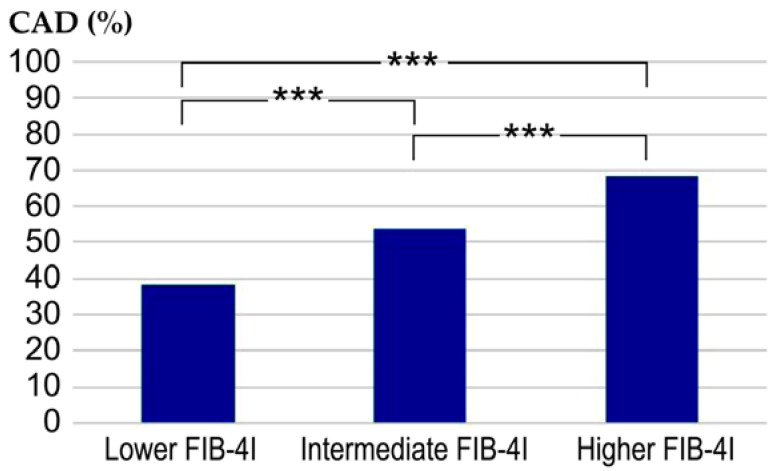
Comparative evaluation of the prevalence of CAD between the three groups. *** *p* < 0.001. Low FIB-4I, ≤1.29; intermediate FIB-4I, 1.30–2.66; high FIB-4I, ≥2.67; CAD, coronary artery disease; FIB-4I, fibrosis-4 index.

Next, we examined the prevalence of CAD in relation to FIB-4I. The values of FIB-4I were classified into three groups based on the severity of liver fibrosis: low risk, intermediate risk, and high risk [9]. Moreover, the patients were also divided into three groups according to their FIB-4I values, namely low FIB-4I (≤1.29), intermediate FIB-4I (1.30–2.66), and high FIB-4I (≥2.67). The prevalences of CAD in the of three groups were 38%, 53.7%, and 68.2%, respectively. Statistically significant differences were observed between these three groups (Figure 2).

**Figure 3:**
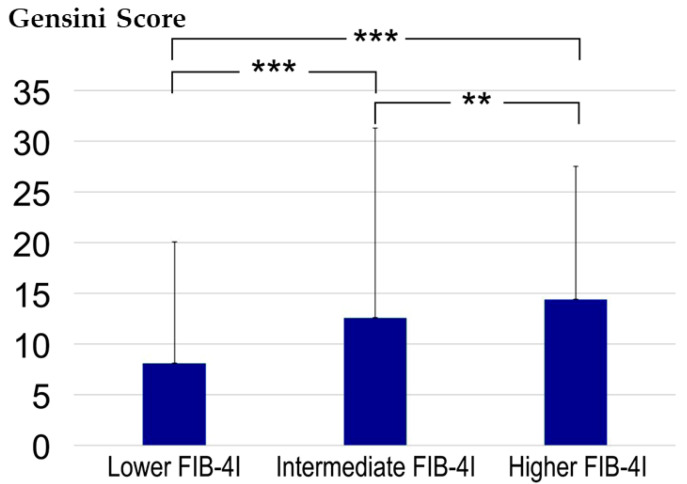
Comparative evaluation of the Gensini score between the three groups. ** *p* < 0.01 and *** *p* < 0.001 according to the Kruskal-Wallis and Steel-Dwass tests. CAD, coronary artery disease; FIB-4I, fibrosis-4 index.

Gensini scores in the low, intermediate, and high FIB-4I groups were 8.1 ± 12.0, 12.6 ± 18.7, and 14.4 ± 13.1, respectively (Figure 3). The differences between the three groups were found to be statistically significant, with *p* < 0.001 for each comparison.

**Figure 4:**
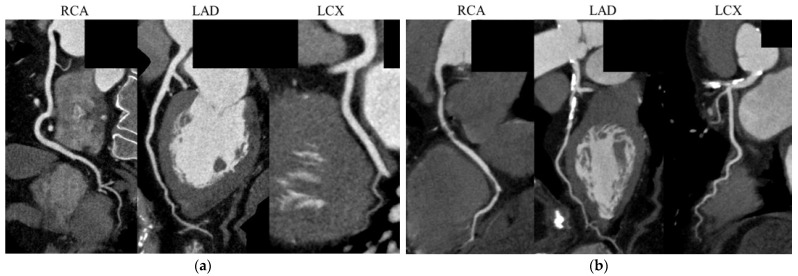
Representative coronary computed tomography angiography images. (**a**) A 66-year-old man with no noticeable CAD, a FIB-4I level of 0.85, and a Gensini score of 0; and (**b**) A 78-year-old woman with a FIB-4I level of 3.13 and a Gensini score of 45, showing multiple coronary artery stenoses. CAD, coronary artery disease; LAD, left anterior descending artery; LCX, left circumflex artery; RCA, right coronary artery.

Figure 4 shows representative CCTA images, including a 66-year-old man with no noticeable CAD, a FIB-4I level of 0.85, and a Gensini score of 0; and a 78-year-old woman with severe CAD, a FIB-4I level of 3.13, and a Gensini score of 45.

As presented in Table 3, a multivariate analysis was conducted regarding the presence of CAD by logistic regression analysis of FIB-4I in addition to conventional risk factors (age ≥ 65 years, male sex, BMI ≥ 25 kg/m^2^, FH, smoking, HTN, DL, DM, CKD, and MetS), which were considered independent variables. Predictors of CAD were age (≥65 years), male sex, HT, DL, and DM along with high FIB-4I level (≥2.67, corresponding to high risk of liver fibrosis) (odds ratio [OR]: 1.92, 95% confidence interval [CI]: 1.30–2.83, *p* = 0.001). Similarly, when low FIB-4 level (≤1.29, corresponding to low risk of liver fibrosis) was incorporated in the analysis instead of high FIB-4 level, low FIB-4 level was also proved to be a predictor of CAD (OR: 0.65, 95% CI: 0.48–0.88, *p* = 0.006). As shown in Table 4, in the HTN group, the FIB-4I levels in the high and low groups were found to be predictors for CAD (OR: 2.01, 95% CI: 1.26–3.21, *p* < 0.001 and OR: 0.65, 95% CI: 0.45–0.94, *p* < 0.022, respectively). However, as shown in Table 5, such a trend was not identified in the non-HTN group (OR: 1.75, 95% CI: 0.84–3.66, *p* = 0.138 and OR: 0.61, 95% CI: 0.36–1.05, *p* = 0.076, respectively).

## 4. Discussion

Our study showed that the FIB-4I level may be a diagnostic indicator for the presence of CAD in patients with HTN who undergo CCTA.

CCTA has become more widely available in several general hospitals and enables accurate noninvasive assessment of coronary artery stenosis [21], calcification [22], and plaque imaging [23]. Although studies have reported an association between FIB-4I and CAD [6,7], to our best knowledge, our study is the first to report a correlation between CAD and the hepatic fibrosis index in patients with high risk for CAD at the time of CCTA from the FU-CCTA registry. We believe that the insights obtained from this study will be useful in daily clinical practice.

The following non-invasive assessments for advanced fibrosis are available for patients with NAFLD: clinical decision support tools (such as the NAFLD fibrosis score, FIB-4I, AST to platelet ratio index [APRI], BMI, AST to ALT ratio, DM index [BARD], and AST to ALT ratio); serum biomarkers (e.g., Enhanced Liver Fibrosis panel, Fibrometer, FibroTest, and Hepascore); and imaging techniques (including transient elastography and magnetic resonance imaging) [9]. FIB-4I provides dual threshold values, wherein scores ≤ 1.29 and ≥2.67 suggest low and high probability of advanced fibrosis, respectively [9]. A recent study comparing multiple risk scores and elastography techniques against hepatic histology demonstrated the superior performance of FIB-4I over other indices such as APRI, BARD, and the AST/ALT ratio [24]. A FIB-4I score of ≥2.67 had a positive predictive value of 80%, whereas a FIB-4I score of ≤1.29 had a negative predictive value of 90%. Although there was no significant difference between the FIB-4I and NAFLD fibrosis scores, the latter requires an insulin resistance index and is consequently more complex to use [24]. Its predictive ability for advanced fibrosis in patients with biopsy-verified NAFLD was equivalent to that of magnetic resonance elastography in Japanese patients [25]. Therefore, we selected FIB-4I out of the various indicators of hepatic fibrosis for this study.

The association between FIB-4I and CAD has been discussed and demonstrated in different groups of patients, including those with NAFLD or DM. Patients with NAFLD who underwent percutaneous coronary intervention showed a close correlation between FIB-4I and the Gensini Score, an indicator of the severity of coronary lesions. This correlation implies the potential for systemic inflammation to mediate both hepatic fibrosis and coronary stenosis [26]. By dividing the patients into three groups on the basis of their FIB-4I levels (low, intermediate, and high), the FIB-4I level increased with the increase in severity of CAD (Figure 3). A notable aspect of our study, in comparison to previous research, is that we included general patients who had undergone CCTA without specifically restricting the participants to those with NAFLD or DM. This suggests that FIB-4I may serve as an indicator for both fibrotic liver disease and coronary arterial damage in patients with risk factors for coronary atherosclerotic diseases.

A prospective study on patients with CAD showed that high FIB-4I values corresponded to increased total mortality and cardiovascular deaths [27]. In terms of overall mortality and cardiovascular death, the high FIB-4I group had hazard ratios (HRs) of 2.84 and 3.34, respectively. This indicates that FIB-4I could be a predictor of clinical outcomes in patients with CAD. FIB-4I has been reported to predict future hepatic and non-hepatic (such as cancers and major adverse cardiac events [MACEs]) events in Japanese patients with NAFLD [28]. The cut-off value, area under the receiver operating characteristic curve (AUROC), sensitivity, and specificity reported were 1.21, 0.739, 92.3%, and 48.2%, respectively. Another study on patients confirmed to have CAD through coronary angiography reported that the NAFLD Fibrosis Score and FIB-4I were not only associated with the severity of coronary artery lesions, but also with future MACEs [29]. In our study, although we did not directly investigate the association between FIB-4I and MACEs, we confirmed a relationship between FIB-4I and the Gensini score. We showed a relationship between the Gensini score and MACEs in our previous study using the FU-CCTA registry [15]. This finding implies a potential correlation between the FIB-4I and MACEs in patients who have undergone CCTA.

FIB-4I can potentially predict the future cardiovascular events in patients with HTN [30]. When using a low FIB-4I value as the reference, the instances of cardiovascular events was reported to be significantly higher in medium and high categories (HRs = 1.88 and 2.98, respectively). In our study, although high and low FIB-4I values were predictive factors for the presence and absence of CAD in patients with HTN, respectively, they did not serve as predictive factors in patients without HTN. Vascular inflammation in patients with HTN appears to be higher in patients with HTN than in non-hypertensive patients. Moreover, the severity of liver fibrosis has also been associated with inflammation. Thus, FIB-4I might only be useful in hypertensive patients who have already undergone a certain degree of atherosclerotic change.

FIB-4I serves as a predictive marker for CAD in patients with metabolically associated fatty liver disease, similar to patients with NAFLD [31]. The AUROC reported was 0.656, and the cut-off for FIB-4I was determined to be 0.85, which yielded a sensitivity of 75% and a specificity of 50%. In our study, the AUROC was 0.61 (95% CI: 0.58–0.64, *p* < 0.001), and the cut-off for FIB-4I was 1.30, which yielded a sensitivity of 74% and a specificity of 43% (figure not shown). The calculated cut-off value of 1.30 for FIB-4I for predicting CAD coincidentally matched the cut-off value for a low fibrosis risk of FIB-4I. In general, CCTA is considered a highly sensitive test and a useful modality for ruling out CAD. This study’s results suggest that if FIB-4I of 1.30 is used as a cut-off value, its good sensitivity might help avoid unnecessary CCTA when FIB-4I is extremely low.

The 2019 European Society of Cardiology guidelines recommend the following approach to the diagnosis of CAD: The pre-test probability and clinical likelihood of CAD should be assessed based on symptoms, physical findings, and basic tests. If the likelihood of CAD is assessed as relatively low, CCTA is recommended for anatomical evaluation, whereas if the likelihood of CAD is relatively high, myocardial perfusion scintigraphy is recommended for the evaluation of physiological ischemia [2]. Because the FIB-4I level can be easily calculated in daily practice, it could serve as a new indicator for estimating the clinical likelihood of CAD. If the FIB-4I level is relatively low, CCTA is not needed. Conversely, if the FIB-4I level is relatively high, conducting myocardial perfusion scintigraphy as an additional test is appropriate. This approach might also have potential benefits from the perspectives of reducing medical radiation exposure and reducing healthcare costs.

This issue requires further discussion. However, some researchers have insisted that this method may not be suitable for primary fibrosis screening in the general population. This is due to the fact that FIB-4I is significantly influenced by age and the AST to ALT ratio. Therefore, there is a necessity for establishing different cut-off values, particularly for older individuals [32]. In this study, we examined the use of FIB-4I in patients with risk factors for atherosclerosis. However, its use in patients without these risk factors remains unexplored and is a subject for future research.

The most critical aspect is the pathogenetic mechanism linking liver fibrosis and CAD. However, the specific mechanisms linking liver fibrosis to CAD have not yet been fully elucidated [33]. One possible mechanism could be systemic inflammation, which is a common factor involved in both the conditions, and it is possible that inflammation drives the progression of each disease state simultaneously or sequentially. Another potential mechanism is oxidative stress, which has been suggested for a similar reason. A third mechanism could be the common risk factors that are inherent in each disease state (HTN, DL, and DM), which could drive the progression of both conditions.

It has also been suggested that fatty liver precedes and subsequently leads to the progression of atherosclerotic diseases [34]. Hepatokines belonging to the category of cytokines are small proteins that are instrumental in transmitting signals between cells [34]. Hepatokines are synthesized and released from the liver [35]. They perform an array of functions within the body, including the control of metabolism, mediation of immune responses, and regulation of inflammation, among other physiological processes [35]. Some of the known hepatokines are Fetuin-A, FGF21, RBP4, and selenoprotein P [35]. Studies have reported that the function of certain hepatokines may be disrupted in conditions such as NAFLD, therefore, playing a role in the development of insulin resistance, DM, and MetS [35].

Age (≥65 years), sex (male), HTN, DL, and DM, in conjunction with the level of FIB-4I, emerged as independent prognostic factors for CAD. Age, sex, HTN, DL, and DM are established critical risk factors for CAD, underscoring the fact that the study participants did not comprise a specific patient cohort. Compared with the non-CAD group, the CAD group was significantly more advanced in terms of age and exhibited higher prevalences of male participants, HTN, DM, DL, MetS, and use of ARB/ACE-Is, CCBs, β-blockers, statins, SUs, and DPP-4Is. The CAD group also presented a significantly lower proportion of females and decreased HDL-C levels compared to the non-CAD group. Given that age, sex, HTN, DM, DL, and MetS are well-recognized crucial risk factors for CAD, it is reasonable that the CAD group showed a higher percentage of medication use for treatment than the non-CAD group.

This study had a few limitations. First, this study was conducted at a single center. Consequently, these findings may not be generalizable to other settings or populations. The use of a multicenter design in future studies could help confirm these findings and improve their generalizability. Second, although CCTA is not a definitive benchmark for evaluation of CAD, recent scholarly investigations have suggested that its sensitivity and specificity are approximately 95% of that demonstrated by invasive coronary angiography for detection of coronary stenosis [10].

## 5. Conclusions

Our results suggest that FIB-4I may serve as a prognostic indicator of the absence or presence of CAD in patients with HTN undergoing CCTA.

## Figures and Tables

**Table 1:** Characteristics, biochemical parameters, and medications in all patients and the CAD and non-CAD groups.

		All Patients	CAD Group	Non-CAD Group	*p*-Value
		(*n* = 1244)	(*n* = 624)	(*n* = 620)	CAD vs. Non-CAD Group
Age, years	Mean (SD)	66 (11)	69 (10)	62 (13)	<0.001
Male sex	%	49	57	41	<0.001
Family history	%	23	22	24	0.42
Smoker	%	34	39	30	0.002
BMI, kg/m^2^	Mean (SD)	24.0 (3.8)	23.9 (3.7)	24.0 (4.0)	0.356
HTN	%	66	76	56	<0.001
SBP, mmHg	Mean (SD)	136 (19)	139 (21)	133 (18)	<0.001
DBP, mmHg	Mean (SD)	78 (13)	79 (13)	78 (13)	0.08
DM	%	25	32	19	<0.001
HbA1c level, %	Mean (SD)	6.0 (0.9)	6.1 (0.9)	5.9 (0.8)	<0.001
FBG level, mg/dL	Mean (SD)	108 (29)	112 (30)	104 (27)	<0.001
DL	%	69	74	64	<0.001
TG level, mg/dL	Mean (SD)	135 (96)	140 (89)	129 (101)	0.027
HDL-C level, mg/dL	Mean (SD)	57 (16)	54 (15)	60 (16)	<0.001
LDL-C level, mg/dL	Mean (SD)	115 (32)	113 (32)	116 (32)	1.0
L/H-C	Mean (SD)	2.2 (0.9)	2.3 (0.9)	2.1 (0.9)	0.056
Non-HDL-C level, mg/dL	Mean (SD)	145 (36)	144 (36)	146 (36)	0.132
CKD	%	29	34	24	<0.001
eGFR, mL/min/1.73 m^2^	Mean (SD)	68 (16)	66 (16)	70 (15)	<0.001
AST level, IU/L	Mean (SD)	27 (17)	27 (18)	26 (16)	0.315
ALT level, IU/L	Mean (SD)	25 (21)	24 (18)	25 (24)	0.837
Plt count, ×10^3^/μL	Mean (SD)	229 (64)	225 (67)	234 (60)	0.007
MetS	%	25	31	20	<0.001
CAD	%	50	–	–	–
VD	Mean (SD)	1.0 (1.1)	1.9 (0.8)	0	<0.001
FIB-4I	Mean (SD)	1.80 (1.22)	1.95 (1.21)	1.65 (1.22)	<0.001
Medications					
ACE-I/ARB	%	36	42	30	<0.001
CCB	%	39	46	31	<0.001
Β-blocker	%	9	11	7	0.003
DU	%	8	10	7	0.086
Statin	%	31	38	25	<0.001
Fibrate	%	1	1	1	0.99
Ezetimib	%	2	2	2	0.573
EPA	%	3	3	2	0.402
SU	%	6	9	4	<0.001
Biguanide	%	6	8	5	0.015
DPP-4I	%	10	13	8	0.003
Insulin	%	2	3	2	0.598

Abbreviations: SD, standard deviation; BMI, body mass index; HTN, hypertension; SBP, systolic blood pressure; DBP, diastolic blood pressure; DM, diabetes mellitus; HbA1c, hemoglobin A1c; FBG, fasting blood glucose; DL, dyslipidemia; TG, triglyceride; HDL-C, high-density lipoprotein cholesterol; LDL-C, low-density lipoprotein cholesterol; L/H-C, low-density lipoprotein cholesterol to high-density lipoprotein cholesterol ratio; CKD, chronic kidney disease; eGFR, estimated glomerular filtration rate; AST, aspartate transaminase; ALT, alanine aminotransferase; Plt, platelet; MetS, metabolic syndrome; CAD, coronary artery disease; VD, number of vessels with significant disease; FIB-4I, fibrosis-4 index; ACE-I, angiotensin-converting enzyme inhibitor; ARB, angiotensin II receptor blocker; CCB, calcium channel blocker; DU, diuretic; EPA, eicosapentaenoic acid; SU, sulfonylurea; DPP-4I, dipeptidyl peptidase-4 inhibitor.

**Table 2:** Differences in FIB-4I values.

FIB-4I				*p*-Value
**All Patients**				
(*n* = 1244) 1.80 ± 1.22				–
**CAD Group**		**Non-CAD Group**		
(*n* = 624) 1.95 ± 1.21		(*n* = 620) 1.65 ± 1.22		<0.001
**HTN and CAD**	**w/o HTN with CAD**			
**Group 1**	**Group 3**			
(*n* = 472) 2.02 ± 1.28	(*n* = 152) 1.72 ± 0.88			0.009
		**HTN w/o CAD**	**w/o HTN and CAD**	
		**Group 2**	**Group 4**	
		(*n* = 345) 1.74 ± 1.34	(*n* = 275) 1.53 ± 1.03	0.014
**HTN Group**		**Non-HTN Group**		
(*n* = 817) 1.90 ± 1.32		(*n* = 427) 1.60 ± 0.98		<0.001
**HTN and CAD**	**HTN w/o CAD**			
**Group 1**	**Group 2**			
(*n* = 472) 2.02 ± 1.28	(*n* = 345) 1.74 ± 1.34			<0.001
		**w/o HTN with CAD**	**w/o HTN and CAD**	
		**Group 3**	**Group 4**	
		(*n* = 152) 1.72 ± 0.88	(*n* = 275) 1.53 ± 1.03	<0.001

Abbreviations: Group 1, HTN and CAD; Group 2, HTN without CAD; Group 3, without HTN with CAD; Group 4, without HTN and CAD; CAD, coronary artery disease; HTN, hypertension; FIB-4I, fibrosis-4 index.

**Table 3:** Predictors of CAD in all patients.

	OR (95% CI)	*p*-Value		OR (95% CI)	*p*-Value
Age (≥65 years)	2.34 (1.81–3.10)	<0.001	Age (≥65 years)	2.12 (1.57–2.85)	<0.001
Sex (Male)	2.02 (1.54–2.65)	<0.001	Sex (Male)	2.05 (1.56–2.69)	<0.001
BMI (≥25 kg/m^2^)	0.82 (0.63–1.07)	0.141	BMI (≥25 kg/m^2^)	0.83 (0.64–1.08)	0.159
FH	1.04 (0.78–1.39)	0.789	FH	1.05 (0.79–1.40)	0.742
Smoker	1.25 (0.94–1.67)	0.118	Smoker	1.24 (0.93–1.64)	0.144
HTN	1.87 (1.42–2.45)	<0.001	HTN	1.88 (1.43–2.47)	<0.001
DL	1.53 (1.16–2.01)	0.003	DL	1.51 (1.15–1.99)	0.003
DM	1.56 (1.17–2.08)	0.003	DM	1.59 (1.19–2.12)	0.002
CKD	1.11 (0.84–1.46)	0.48	CKD	1.11 (0.84–1.46)	0.474
MetS	1.11 (0.81–1.53)	0.513	MetS	1.08 (0.78–1.48)	0.646
FIB-4I (≥2.67)	1.92 (1.30–2.83)	0.001	FIB-4I (≤1.29)	0.65 (0.48–0.88)	0.006

Abbreviations: BMI, body mass index; FH, family history of cardiovascular disease; HTN, hypertension; DL, dyslipidemia; DM, diabetes mellitus; CKD, chronic kidney disease; MetS, metabolic syndrome; FIB-4I, fibrosis-4 index; OR, odds ratio; CI, confidence interval.

**Table 4:** Predictors of CAD in hypertensive patients.

	OR (95% CI)	*p*-Value		OR (95% CI)	*p*-Value
Age (≥65 years)	2.35 (1.68–3.27)	<0.001	Age (≥65 years)	2.11 (1.47–3.04 )	<0.001
Sex (Male)	1.46 (1.04–2.03)	0.027	Sex (Male)	1.49 (1.07–2.08)	0.019
BMI (≥25 kg/m^2^)	0.81 (0.59–1.11)	0.185	BMI (≥25 kg/m^2^)	0.80 (0.58–1.10)	0.172
FH	0.80 (0.56–1.14)	0.218	FH	0.80 (0.56–1.13)	0.204
Smoker	1.44 (1.02–2.05)	0.041	Smoker	1.40 (0.99–1.99)	0.059
DL	1.70 (1.20–2.41)	0.003	DL	1.69 (1.19–2.39)	0.003
DM	1.75 (1.25–2.46)	0.001	DM	1.79 (1.28–2.51)	<0.001
CKD	1.04 (0.76–1.44)	0.799	CKD	1.05 (0.76–1.45)	0.771
MetS	1.09 (0.77–1.54)	0.623	MetS	1.05 (0.75–1.49)	0.765
FIB-4I (≥2.67)	2.01 (1.26–3.21)	<0.001	FIB-4I (≤1.29)	0.65 (0.45–0.94)	0.022

Abbreviations: BMI, body mass index; FH, family history of cardiovascular disease; DL, dyslipidemia; DM, diabetes mellitus; CKD, chronic kidney disease; MetS, metabolic syndrome; FIB-4I, fibrosis-4 index; OR, odds ratio; CI, confidence interval.

**Table 5:** Predictors of CAD in non-hypertensive patients.

	OR (95% CI)	*p*-Value		OR (95% CI)	*p*-Value
Age (≥65 years)	2.75 (1.67–4.53)	<0.001	Age (≥65 years)	2.34 (1.36–4.04)	0.002
Sex (Male)	3.95 (2.40–6.49)	<0.001	Sex (Male)	3.96 (2.41–6.51)	<0.001
BMI (≥25 kg/m^2^)	0.87 (0.53–1.43)	0.592	BMI (≥25 kg/m^2^)	0.92 (0.56–1.52)	0.743
FH	1.71 (1.03–2.85)	0.037	FH	1.79 (1.08–2.97)	0.025
Smoker	1.02 (0.62–1.67)	0.948	Smoker	1.03 (0.62–1.70)	0.910
DL	1.20 (0.76–1.90)	0.433	DL	1.18 (0.74–1.86)	0.485
DM	0.97 (0.51–1.84)	0.927	DM	0.98 (0.51–1.87)	0.945
CKD	1.39 (0.79–2.47)	0.255	CKD	1.37 (0.77–2.43)	0.285
MetS	1.58 (0.55–4.52)	0.395	MetS	1.56 (0.55–4.44)	0.405
FIB-4I (≥2.67)	1.75 (0.84–3.66)	0.138	FIB-4I (≤1.29)	0.61 (0.36–1.05)	0.076

Abbreviations: BMI, body mass index; FH, family history of cardiovascular disease; DL, dyslipidemia; DM, diabetes mellitus; CKD, chronic kidney disease; MetS, metabolic syndrome; FIB-4I, fibrosis-4 index; OR, odds ratio; CI, confidence interval.

## Data Availability

Data supporting the findings of this investigation can be obtained from the corresponding author via appropriate request.
